# Cone Beam CT Analysis of Haller Cells: Prevalence and Relationship with Orbital Floor Dehiscence

**DOI:** 10.1155/2023/5200152

**Published:** 2023-01-31

**Authors:** Mahkameh Moshfeghi, Hamidreza Dehini, Mitra Ghazizadeh Ahsaie

**Affiliations:** ^1^Department of Oral and Maxillofacial Radiology, School of Dentistry, Shahid Beheshti University of Medical Sciences, Tehran, Iran; ^2^School of Dentistry, Shahid Beheshti University of Medical Sciences, Tehran, Iran

## Abstract

**Materials and Methods:**

CBCT images of 120 patients were interpreted in coronal plane for the presence of Haller cells and orbital floor dehiscence. The prevalence of Haller cell, presence of dehiscence, unilateral, or bilateral frequency were assessed. In addition, the size was categorized in three groups of small, medium, and large. Chi-square and Cochran–Mantel–Haenszel tests were used for statistical analysis of the data, and *p* < 0.05 was considered to be significant.

**Results:**

A total of 51 male and 69 female with mean ± SD age of 38.84 ± 68.14 were assessed. The overall prevalence of Haller cells was 56.7%, of which 44 (64.7%) were unilateral and 24 were bilateral (35.3%). The majority of the cells (70.7%) were seen in medium (2–4 mm) sized. There was a significant association between Haller cells and orbital floor dehiscence (*p* ≤ 0.001).

**Conclusion:**

The prevalence of Haller cells was remarkably high and the presence of Haller cells was strongly associated with ipsilateral orbital floor dehiscence. Based on the findings of this study, CBCT can be useful in delineation of the bony anatomy of sinonasal complex.

## 1. Introduction

One of the problems in oral and maxillofacial procedures is having many different anatomical features in different patients. Haller cells are one of these normal variations of paranasal and nasal areas, which are related to some symptoms and diseases [1, 2]. These cells arise from anterior ethmoid air cells and are located in the sinus floor, medial orbital floor, lateral to the maxillary infundibulum, and in the most inferior part of lamina papyracea [1, 3]. Haller cells were first introduced by a Swiss anatomist named “Albert Von Haller” in 1756. Other names for Haller cell are orbitoethmoidal cell and maxilloethmoidal cell [1, 4]. In addition to orofacial pain and sinusitis, Haller cells can cause other maladies including nasal congestion, incomplete nasal breathing, headache, chronic coughs, and mucocele [1–3]. Haller cell position may lead to disruption of the normal pattern of mucocilliary flow that causes recurrent maxillary sinusitis [4, 5]. These cells are discovered in paranasal CT examinations by accident [5, 6]. The prevalence of Haller cells in CT examinations has a wide range [1, 3, 7]. Hui et al. indicated that the prevalence of Haller cell is 29.5% and there was no significant relation between the presence of Haller cell and maxillary sinus pathologies [8]. Some studies showed a significant relationship between Haller cell size (>3 mm) and orbital floor dehiscence; nevertheless, there is no definite information on this matter [2, 6]. In 2013, Mathew et al. studied CBCTs of 50 patients, which showed a 60% prevalence for Haller cells. There was no significant relation between the existence and size of Haller cell, size of maxillary ostium and maxillary sinusitis; however, there was a significant relation between Haller cell and dehiscence of orbital floor [6]. In 2013, Khayam et al. studied panoramic radiographic images of 200 patients to determine the existence of Haller cells. The prevalence of Haller cells was 32.5% [9]. In 2012, Raina et al. surveyed panoramic radiography of 600 patients, and the prevalence of Haller cell was 16% [1]. In 2010, Valizadeh also studied 310 panoramic radiographic images, and the prevalence of Haller cells was 37%, which showed that Haller cells can be a common normal landmark in panoramic radiography [10]. In 2005, Lerdlum and Vachiranubhap studied CT images of 133 patients to determine the prevalence of sinus anatomical variations. Haller cell was the second prevalent anatomical variation (9.4%); yet in this study, only agger nasi cells (anterior ethmoidal cells) had a significant relation with sinusitis [4]. Haller cells can limit the accessibility to the maxillary sinus and anterior ethmoidal cells in endonasal surgeries; thus, surgeons must be informed about these anatomical variations, which increase the risk of complications after surgeries [1]. Due to the limitations of previous studies and lack of evidence in Haller cell topic, the aim of this study was to determine the prevalence of Haller cells and its relationship with orbital floor dehiscence in CBCT images.

## 2. Materials and Methods

This cross-sectional study was approved by the Research Committee of Shahid Beheshti University of Medical Sciences and it was conducted in accordance with the Declaration of Helsinki and its subsequent revisions. The study was conducted in accordance with the STROBE statement. 120 samples were selected by the simple sampling method from referred patients to radiology department of dental school. The CBCT scans had been requested for purposes not related to this study from 2018 to 2019. The CBCT scans had been obtained by the New Tom VGI CBCT scanner (Quantitative radiology, Verona, Italy) in two centers with the exposure settings of 110 kVp and 3.3–20 mA. Patients were included if they needed CBCT for different purposes (e.g., dental implants, jaw lesions, TMJ, and orthodontic evaluations), and they were excluded if they had a history of tumor, surgery, sinus problems, sinonasal polyposis, trauma of the midface, and if younger than 16. The images were further analyzed by NNT software in coronal plane by an experienced oral and maxillofacial radiologist.

**Figure 1:**
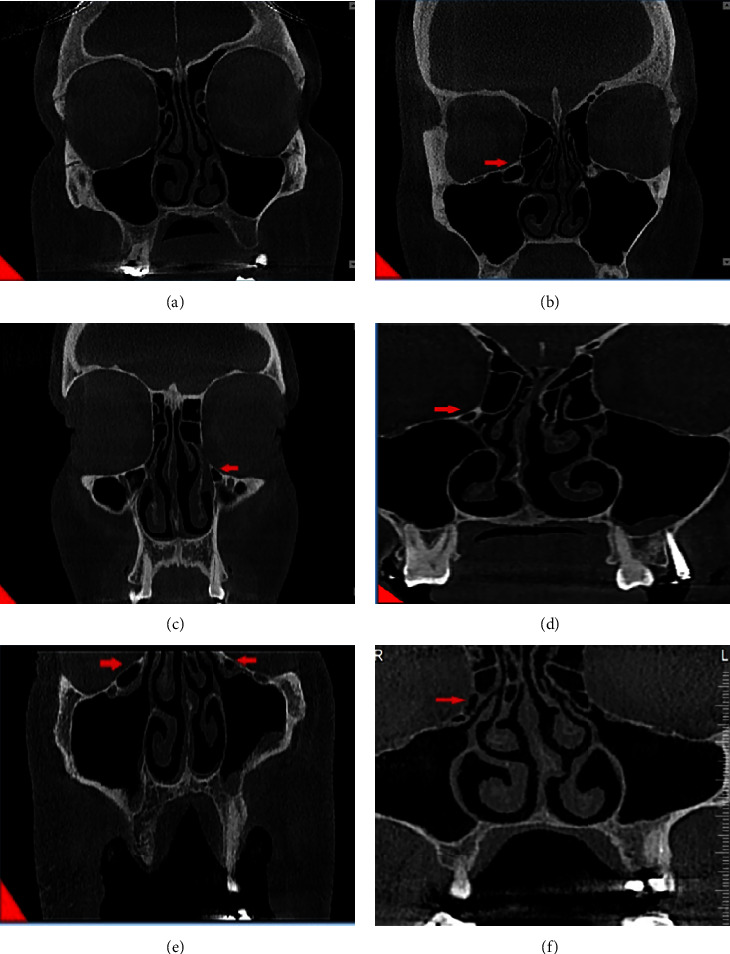
Detection of Haller cell in coronal view CBCT. (a) No Haller cell is detected. (b) Large Haller cell on the right side. (c) Medium sized Haller cell on the left. (d) Small-sized Haller cell on the right. (e) Bilateral presence of Haller cell with two different sizes. (f) Dehiscence of inferomedial border of orbit due to the presence.

A meticulous criteria for defining Haller cells as air cells was used, for any size located along medial portion of the orbital floor and/or lamina papyracea inferior to the bulla ethmoidalis and continuous with ethmoid capsula. The continuity with ethmoid capsula distinguishes Haller cells from infraorbital recess of maxillary sinus. Haller cell size was measured by its maximum mediolateral dimension. Maxillary ostium size is the distance between the most medial part of Haller cell and the uncinate process. Ostium and Haller cells based on size, are divided into 3 groups: small (less than 2 mm), medium (2–4 mm), and large (more than 4 mm). Infraorbital dehiscence is defined as the loss of bone density, and when the difference between dehiscence and thin bone wall is not recognizable, dehiscence diagnosis is acceptable (Figure 1).

All collected data were executed by using the SPSS software (version 19), and the association between them was tested by the chi-squared test and Cochran–Mantel–Haenszel test.

## 3. Results

In this study, 120 patients (42.5% male and 57.5% female) were included from ages 18 to 79 with the average of 38.84. There was no significant relation between Haller cells and gender (*p*=0.682). Sixty-eight patients had Haller cells in their CBCTs, so the prevalence of Haller cells in this population was 56.7%, which included 64.7% unilateral and 35.3% bilateral. Both men and women were the same in the number of unilateral Haller cells, but in men, unilateral Haller cells were three times more than bilateral ones. Also, bilateral cases were twice in females. There was no significant relation between unilateral/bilateral and gender/age (*p*=0.186/*p*=0.419). Haller cells were categorized into three groups according to mediolateral dimensions: (a) small: less than 2 mm, (b) medium: 2–4 mm, and (c) large: more than 4 mm. Twenty percent of Haller cells were small, 70.67% were medium, and 9.33% were large, so the most prevalent size of Haller cells was medium (*p* ≤ 0.001). Even though the prevalence of Haller cells is various in different ages, we can assume all ages the same (*p*=0.282). Furthermore, there is no relation between size of Haller cells and gender/age (*p*=0.414/*p*=0.668).

From 68 patients having Haller cells, 11 cases had orbital floor dehiscence. Fifty-two participants did not have Haller cells, also did not have orbital floor dehiscence. So, in this study, coexistence of Haller cells and orbital floor dehiscence is confirmed.

## 4. Discussion

This study estimated the prevalence of Haller cells in CBCT images almost high and about 56.7%. In some studies, an extremely variable range (2%–70.3%) has been reported for the prevalence of Haller cells [8, 11–25]. Mathew et al. [6] reported a 60% prevalence for Haller cells and Khojastepour et al. [26] reported 68%, which are close to our study. Alkire and Bhattacharyya [13] also reported a 70.3% prevalence for Haller cells. This variability can be due to variation in subjects' race, age, sample size, observer's judgement regarding the presence of Haller cells in images, and different definitions for Haller cells in different studies. On the other hand, imaging technique also changes the results. Due to CBCT being a volumetric imaging technique, all the Haller cells in any size get captured; on the contrary, in multislice CT scans, small Haller cells could easily be missed in the interslice intervals [6]. The high percentage of Haller cells in this study can represent the high sensitivity of CBCT scan in the detection of small delicate bony structures.

In this study, Haller cells were present unilaterally with statistical significance (64.7%). This finding is compatible with a large number of the previous studies [2–5, 7, 9–24, 26–39].

In Mathew's study, Haller cells were mostly present bilaterally, which was not statistically significant. The difference in the results can be due to the difference in the population and smaller sample size in Mathew's study (*n* = 50) than the present study (*n* = 120). Our study showed that the prevalence of Haller cell in women is slightly higher than in men. However, this difference is not statistically significant. Ozcan et al. also indicated that the prevalence of Haller cell is three times higher in female than in male [25]. These findings are consistent with the results of Khojastepour et al. [26], Raina et al. [1], and Basic et al.'s [40] studies.

The most prevalent Haller cells observed in our study were medium sized (2–4 mm) with a significant difference. In the study of Dhillon and Kalra [32], about 51% of cases were large (>4 mm). The difference between these two studies can be justified by the variation in the ethnic characteristics of the populations studied and the sample size.

In this study, more than 55% of positive cases were under 40 years old. However, the difference in the distribution of Haller cell prevalence by age was not statistically significant. In Raina et al.'s study, 64.6% of Haller cells were observed in subjects from ages 18 to 30, which is consistent with our study results [1].

Various studies have assessed the relationship between presence of Haller cell and maxillary sinus drainage and pathologies such as sinusitis [41–44]. Although presence of Haller cells may interfere with normal sinus drainage, Suzuki-Yamazaki performed a successful sinus lift procedure in a patient with large Haller cell [41]. To the best of our knowledge, this is one of the first studies assessing the relationship of Haller cell size and orbital floor dehiscence in Iranian population. This study confirmed the existence of a significant relationship between the presence of Haller cells and dehiscence of orbital floor (*p*=0.002). Lack of bone density and presence of only a mucoperiosteal lining that separates Haller cell from orbital space were considered as dehiscence. Diagnosis of this issue in CBCT images is really important because the presence of orbital floor dehiscence predisposes orbital space to Haller cell diseases or makes it vulnerable in osteomeatal complex surgery.

Seberchets et al. published an article in 2000 containing three case reports of unilateral orbital cellulitis caused by inflammation of the Haller cells [45]. They declared that any pathologic lesion related to Haller cells should be considered as a potential for unilateral eye cellulitis. Given that there is no lymphatic drainage system in the eye, they propounded a hypothesis that infection spreads through orbital floor dehiscence, lamina papyracea, or sutures of the medial part of orbital floor. Mathew et al.'s study also showed a significant relationship between Haller cells and orbital floor dehiscence; both of these results support our study (3).

So according to this study, we suggest that anytime an inflamed Haller cell is observed in CBCT. Simultaneous presence of orbital floor dehiscence should be expected.

## 5. Conclusion

This study estimated the prevalence of Haller cells in CBCT high (56.7%) and noticeable and also showed that statistically, there is a significant relationship between Haller cells and orbital floor dehiscence. It can be concluded that CBCT can be a useful imaging modality for evaluating the anatomical aspect of sinonasal bone complex due to its high accuracy and lower radiation dose.

## 6. Limitations and Suggestions

### 6.1. Limitation

Due to the nature of this study (in vitro) and method of collecting the images (CBCTs stored in university's archive), there was no ethical limitation.

### 6.2. Recommendations

For increasing the accuracy and efficiency of the study, we suggest a larger population with more samples available.

Different resolutions should be used in CBCT imaging for better diagnosis of Haller cells and orbital floor dehiscence.

## Data Availability

The datasets used and/or analyzed during the current study are available from the corresponding author upon request.
